# Impact of the Genome on the Epigenome Is Manifested in DNA Methylation Patterns of Imprinted Regions in Monozygotic and Dizygotic Twins

**DOI:** 10.1371/journal.pone.0025590

**Published:** 2011-10-03

**Authors:** Marcel W. Coolen, Aaron L. Statham, Wenjia Qu, Megan J. Campbell, Anjali K. Henders, Grant W. Montgomery, Nick G. Martin, Susan J. Clark

**Affiliations:** 1 Epigenetics Research Group, Cancer Program, Garvan Institute of Medical Research, Sydney, Australia; 2 Department of Human Genetics, Nijmegen Centre for Molecular Life Sciences, Radboud University Nijmegen Medical Centre, Nijmegen, The Netherlands; 3 Genetic and Molecular Epidemiology Laboratories, Queensland Institute of Medical Research, Brisbane, Queensland, Australia; 4 St. Vincent's Clinical School, Faculty of Medicine, University of New South Wales, Sydney, Australia; University of Illinois at Chicago, United States of America

## Abstract

One of the best studied read-outs of epigenetic change is the differential expression of imprinted genes, controlled by differential methylation of imprinted control regions (ICRs). To address the impact of genotype on the epigenome, we performed a detailed study in 128 pairs of monozygotic (MZ) and 128 pairs of dizygotic (DZ) twins, interrogating the DNA methylation status of the ICRs of *IGF2*, *H19*, *KCNQ1*, GNAS and the non-imprinted gene *RUNX1*. While we found a similar overall pattern of methylation between MZ and DZ twins, we also observed a high degree of variability in individual CpG methylation levels, notably at the *H19*/*IGF2* loci. A degree of methylation plasticity independent of the genome sequence was observed, with both local and regional CpG methylation changes, discordant between MZ and DZ individual pairs. However, concordant gains or losses of methylation, within individual twin pairs were more common in MZ than DZ twin pairs, indicating that *de novo* and/or maintenance methylation is influenced by the underlying DNA sequence. Specifically, for the first time we showed that the rs10732516 [A] polymorphism, located in a critical CTCF binding site in the *H19* ICR locus, is strongly associated with increased hypermethylation of specific CpG sites in the maternal *H19* allele. Together, our results highlight the impact of the genome on the epigenome and demonstrate that while DNA methylation states are tightly maintained between genetically identical and related individuals, there remains considerable epigenetic variation that may contribute to disease susceptibility.

## Introduction

Genetic polymorphisms are heritable alterations in the DNA sequence and contribute to phenotypic variation, and sometimes to disease susceptibility, through effects on gene expression and function. Twin studies however, demonstrate that genetic polymorphisms alone are often insufficient to cause phenotypic variance and disease susceptibility, and other factors are required, including possible epigenetic factors. In contrast to genetic alterations, an epigenetic alteration is defined as a heritable change in gene expression that does not involve a change in the DNA sequence. Epigenetic mechanisms play an essential role in eukaryotic gene regulation by changes in DNA methylation and modification of chromatin structure, which in turn modulate gene expression. However, how the sequence of the genome and single nucleotide polymorphisms (SNPs) may impact on the epigenome, in particular in modulating regional specific DNA methylation and gene expression patterns is still largely unknown.

**Figure 1:**
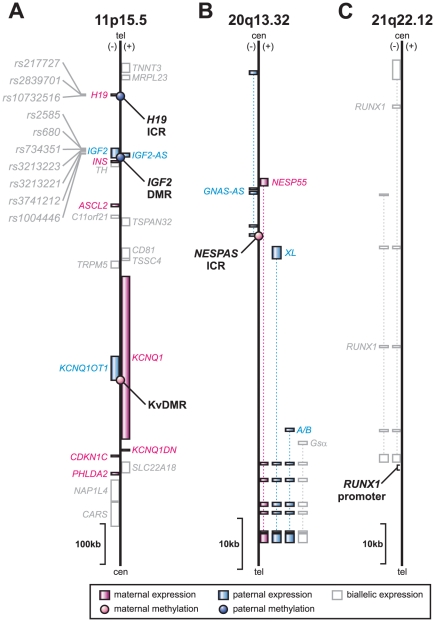
Genomic regions interrogated. Four imprinted regions were tested for their DNA methylation levels: (A) three are located in close proximity of each other on 11p15.5: *H19*-ICR, *IGF2*-DMR and KvDMR, and (B) one is located on 20q13.32: *NESPAS*-ICR. (**C**) As a non-imprinted region the *RUNX1* promoter was interrogated. Features on the (+) and (−) strand are shown to the right or left of the line, respectively. The imprinting status of the genes is shown in pink (maternally expressed), blue (paternally expressed), and white (biallelic expression or imprinted expression not known). Intronic regions are indicated as dotted lines. Imprinting control regions and differentially methylated regions (ICR and DMR, respectively) are marked by circles coloured according to the parental origin of the imprint (tel: telomeric end; cen: centromeric end [adapted from 2], [43]). Single nucleotide polymorphisms interrogated in this study are also indicated on 11p15.5.

Imprinted genes are transcribed from only one of the parental chromosomes. To date, about 80 imprinted genes have been identified in mammals that display parental specific expression. Although dispersed throughout the genome, imprinted genes are often found in clusters, which are regulated via a central control element, also known as imprinting control region (ICR). The epigenetic state of an ICR, and in particular the DNA methylation level has been shown to be crucial for maintaining the imprinted state of the genes in a cluster [1]. As the control regions display allele specific DNA methylation they are also referred to as differentially methylated regions (DMRs). The best documented imprinting cluster harbours the genes *IGF2* and *H19*, and is located on chromosome 11p15.5 in humans (Figure 1A) [2]. These adjacent genes are reciprocally imprinted; the potential growth factor *IGF2* is paternally expressed, whereas for the noncoding RNA *H19*, the maternal allele is active [3], [4]. Extensive evidence shows the imprinting status of the *IGF2*/*H19* locus is regulated by an ICR upstream of *H19* [5] that displays parent-of-origin-dependent (paternal) methylation and contains binding sites for the zinc-finger CCCTC-binding factor, known as CTCF. Other elements within the *IGF2*/*H19* locus have also been reported to be involved in the regulatory mechanism of the region [6], [7], [8]. Importantly, the methylation status of one of these elements (*IGF2*-DMR; located in the last exon of *IGF2* and normally only methylated on the paternal allele) has been reported to be associated with the risk of developing human colorectal cancer in some studies [9] but not in others [10]. A second imprinting domain is located centromeric to the *IGF2*/*H19* locus, referred to as *KCNQ1* (Figure 1A). This domain contains several maternally expressed genes and a single paternally expressed transcript, *KCNQ1OT1*. The promoter of *KCNQ1OT1* is contained within intron 10 of the *KCNQ1* gene and harbours the imprinting control region KvDMR. KvDMR is methylated on the maternal allele and has been implicated in the manifestation of parent-of-origin-specific expression of the genes in the locus. Moreover, disruption of the differential methylation of KvDMR is observed in over half of the cases of the congenital overgrowth disorder called Beckwith-Wiedemann syndrome (BWS) [11], [12], [13], as well as in colorectal cancer tissue specimens [14]. Another example of clustering of imprinted genes in the human genome is the *GNAS* locus on 20q13.32. *GNAS* is a complex locus, which gives rise to alternatively spliced isoforms that show maternal-, paternal- and biallelic expression as well as a noncoding antisense transcript (Figure 1B) [15]. The principal control region *NESPAS*-ICR is methylated on the maternal allele and contains the promoter for the antisense transcript [16]. Disruption of the (regulation of the) *GNAS* locus can result in various human disorders, including pseudohypoparathyroidism and some tumours of different origin [17].

It is now well accepted that the DNA methylation status of imprinting control regions plays a critical role in the regulation of imprinted genes. However, it is still poorly understood how variable the DNA methylation levels at these functionally important regions are between individuals. In addition, it is not known to what extent the epigenome is influenced by an individual's genome sequence, whether DNA methylation variability is restricted to localised regions or if the variability is more generally regulated across chromosomes. To answer these questions, we examined in detail the DNA methylation status of four imprinting control regions (*H19*-ICR, *IGF2*-DMR, KvDMR and *NEPAS*-ICR) located both in clusters and on different chromosomes in a sample of 128 pairs of monozygotic and 128 pairs of dizygotic twins, to address potential in *cis* (at localised regions) and/or in *trans* (between regions or across chromosomes) methylation variance in identical and related individuals. We also included a DNA methylation analysis of the *RUNX1* promoter, a biallelically expressed gene that has shown evidence of sporadic methylation in normal peripheral blood cells [18] to gauge the level of variation relative to variation at imprinted loci (Figure 1C). Finally, ten SNPs within the *IGF2*/*H19* locus were examined to investigate any potential link between genotype and epigenotype (Figure 1A).

We show that DNA methylation levels at critically important imprinted regions differed considerably, even within identical twin pairs. The variation is typically confined to localised regions, and occurs randomly between the regions. Importantly, we propose that this variability allows for possible phenotypic diversity and variability in disease susceptibility. Interestingly, there appears to be a genetic contribution to the DNA methylation level at the *H19* imprinting control region. Here, we find that a single nucleotide polymorphism appears to have substantial impact on the DNA methylation status of the region, but only if inherited on the maternal allele. We speculate that this polymorphism may directly affect the binding affinity of the insulator protein CTCF to this region.

## Materials and Methods

### Subjects

The twins analysed are part of a study on moliness and cognitive function [19], [20], [21] and comprised 512 adolescent twins (70 female and 58 male monozygotic twin pairs; 25 female, 29 male and 74 opposite sex dizygotic twin pairs), with a mean age of 14.15 years (SD = 2.46; range 12–22.85). The samples are predominantly (>95%) of northern European origin (mainly Anglo-Celtic). Zygosity of the twins was confirmed using microsatellite repeat marker testing. All cases and controls gave informed consent to participation in this study, and the study protocol was approved by the Queensland Institute of Medical Research Human Research Ethics Committee, (Ethics approvals P193 and P455).

### DNA methylation assay design

MassCleave assays against the genomic regions of interest were designed and tested using the *AmpliconReport* R-script we described previously [22]. The analysed regions encompassed the imprinted regions *H19*-ICR [23], *IGF2*-DMR [9], KvDMR [13], [14] and *NESPAS*-ICR [16], and the promoter of the non-imprinted *RUNX1* gene (Figure S1).

### Genomic bisulphite treatment

DNA methylation measurements were performed on genomic DNA extracted from whole blood. Bisulphite treatment was carried out using the EZ-96 DNA Methylation-Gold Kit (Zymo Research: Cat No. D5008) according to the manufacturer's instructions. In brief, 500 ng DNA was used in the bisulphite reaction and incubated for 8 hours at 55°C. After desulfonation and clean up, the bisulphite treated DNA was resuspended in 50 µl of which 2 µl was used in each PCR.

### PCR-tagging, in vitro transcription and mass spectrometry analysis

The target regions were amplified in triplicate using the primer pairs and annealing temperatures (Ta) described in Table S1. The MassCleave methylation analysis was performed as described previously [22]. In brief, the PCR reactions were carried out in a total volume of 5 µl using 200 nM forward and reverse primer, 200 µM dNTPs, 1.5 mM MgCl_2_ 1∶100,000 dilution of SYBR Green (Invitrogen) and 0.35 U Platinum *Taq* DNA polymerase (Invitrogen) in 1× PCR buffer without magnesium. PCR success was determined via melt curve analysis (ABI PRISM® 7700). Unincorporated dNTPs were dephosphorylated by incubation at 37°C for 20 min in the presence of 1.7 µl H_2_O and 0.3 U Shrimp Alkaline Phosphatase (SAP), followed by a heat-inactivation for 5 min at 85°C. The triplicate PCR samples after SAP-treatment were pooled and of this pool, two microliters were used in a 7 µl transcription reaction, containing 3.14 mM DTT, 2.5 mM dCTP, 1 mM rUTP, 1 mM rGTP, 1 mM rATP, 20 U T7 R&DNA polymerase and 0.09 mg/ml RNase A in 0.64× T7 polymerase buffer (all reagents from SEQUENOM, San Diego). Transcription and digestion were performed in the same step at 37°C for 3 hours. After the addition of 20 µl H_2_O, conditioning of the phosphate backbone prior to MALDI-TOF MS was achieved by the addition of 6 mg CLEAN Resin (SEQUENOM, San Diego). Twenty-two nanoliters of the cleavage reactions were robotically dispensed onto silicon chips preloaded with matrix (SpectroCHIPs; SEQUENOM, San Diego). Mass spectra were collected using a MassARRAY mass spectrometer (SEQUENOM).

### Calculation of methylation ratios

Calculation of the DNA methylation levels was performed using the R-script *Analyze Sequenom Function* (ASF) which we described previously [22]. All statistical calculations were carried out using either Stata 9 (StataCorp LP, Texas, USA) or the free ‘R’ software package for statistical computing (http://www.R-project.org).

### Clonal analysis of DNA methylation status

For clonal analysis, three independent PCR reactions were performed and products pooled to ensure a representative methylation profile. PCR products were purified using the Wizard PCR DNA purification system and cloned into the pGEM-T-Easy Vector (Promega) using the Rapid Ligation Buffer System (Promega). Individual clones were purified and sequenced and the methylation status for each CpG site was determined.

### Genotyping

The SNPs rs217727, rs2839701, rs2585, rs680, rs734351, rs3213223, rs3213221, rs3741212 and rs1004446 residing in the *IGF2*/*H19* locus were genotyped in multiplex assays designed using the Sequenom MassARRAY Assay Design software (version 3.0). SNPs were typed using Sequenom iPLEX chemistry on a MALDI-TOF Compact Mass Spectrometer (SEQUENOM). The 2.5 µl PCR reactions were performed in standard 384-well plates using 12.5 ng genomic DNA, 0.8 unit of *Taq* polymerase (HotStarTaq; QIAGEN, Australia), 500 µmol of each dNTP, 1.625 mM of MgCl_2_, and 100 nmol of each PCR primers (Bioneer, Korea). Standard PCR thermal cycling conditions and post-PCR extension reactions were carried out as described previously [24]. The iPLEX reaction products were desalted by diluting samples with 15 µl of water and adding 3 µg of resin. The products were spotted on a SpectroChip (SEQUENOM), and data were processed and analyzed by MassARRAY TYPER 3.4 software (SEQUENOM). The rs10732516 and rs45596642 genotypes were determined from the MassARRAY methylation analyses profiles as described previously [22].

### Statistical analysis

The quantitative methylation of each CpG unit measured by the MALDI-TOF-MS assay was filtered to exclude poor-quality data. Only CpG units with values in at least 75% of the 512 samples were selected. The results for the CpG unit harbouring the rs10732516 SNP in the *H19*-ICR were removed from the DNA methylation analysis as this polymorphism affected the methylation call in the assay. This resulted in the analysis of 83 CpG sites divided over 54 CpG units and originating from five regions in the genome. Correlations between the methylation levels of different CpG sites were calculated using the nonparametric Spearman correlation test (two-tailed, *P*-value<0.05). The non-parametric Kruskal-Wallis test, followed by a Dunn's multiple comparison test was used to determine whether a statistically significant difference was observed between the discordance levels in MZ, DZ and non-related individuals. The group of non-related individuals was created by randomly mixing MZ or DZ individuals that were not of the same family within at least two generations. Significant differences between MZ and DZ twins in the presence of (concordant) loss or gain of methylation (LOM or GOM, respectively) were tested using the chi square test.

### CTCF binding analysis

Publically available ChIP-seq data from the ENCODE Chromatin Group at the Broad Institute [25] was downloaded for all available normal cell lines (HMEC [human mammary epithelial cells], HSMM [normal human skeletal muscle myoblasts], HUVEC [human umbilical vein endothelial cells], NHEK [normal human epidermal keratinocytes], NHLF [normal human lung fibroblasts] and the H1 human embryonic stem cell line). The Position Weight Matrix (PWM) for the CTCF binding sequence was obtained from the JASPAR database [26]. Mean enrichment scores near all sequences that matched the PWM with at least 80% sequence match were calculated on all autosomes and the X chromosome in all 6 cell lines (−500 to +500 bp relative to the CTCF binding site; n = 52,588 sites). Next, the cytosine at position 6 of the PWM for CTCF was replaced with a thymidine and the mean enrichment score was calculated again (n = 32,334 sites). To account for differences in the number of reads between samples, the comparison of mean enrichment scores was performed directly within each cell line.

## Results

### DNA methylation variance at *H19*-ICR, *IGF2*-DMR, KvDMR, *NESPAS*-ICR and the *RUNX1* promoter in MZ and DZ twins

**Figure 2:**
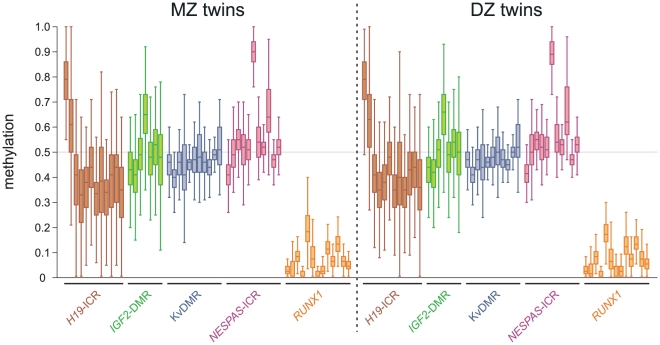
DNA methylation levels at four imprinted loci and the *RUNX1* promoter in MZ and DZ twins. Box plot results of the DNA methylation levels are shown per CpG unit as determined by MALDI-TOF mass spectrometry. Each box plot shows the distribution of DNA methylation in 256 monozygotic (MZ) or dizygotic (DZ) individuals (left or right panel, respectively; brown: *H19*-ICR; green: *IGF2*-DMR; blue: KvDMR; pink: *NESPAS*-ICR; orange: *RUNX1*).

We performed a detailed DNA methylation analysis of four imprinted control regions (ICRs: *H19*-ICR, *IGF2*-DMR, KvDMR and *NESPAS*-ICR) and of the *RUNX1*/*AML1* promoter interrogating 14, 8, 15, 17 & 29 CpG sites respectively (Figure S1) from whole blood DNA from 256 pairs of adolescent twins. We found that MZ and DZ individuals have remarkably similar DNA methylation levels and as expected, the median methylation levels of the imprinted regions were around 50% with some exceptions for individual CpG units that displayed elevated levels (Figure 2). In contrast, the *RUNX1* promoter only contained low levels of DNA methylation with median levels ranging from 0% to 18% (Figure 2). Notably, for both the MZ and DZ pairs we found that *H19*-ICR and *IGF2*-DMR DNA methylation levels displayed a considerable degree of variation, far greater than KvDMR, *NESPAS*-ICR or the *RUNX1* promoter (average interquartile ranges for *H19*-ICR, *IGF2*-DMR, *NESPAS*-ICR, KvDMR and *RUNX1* are 0.12, 0.12, 0.05, 0.05 and 0.06, respectively).

**Figure 3:**
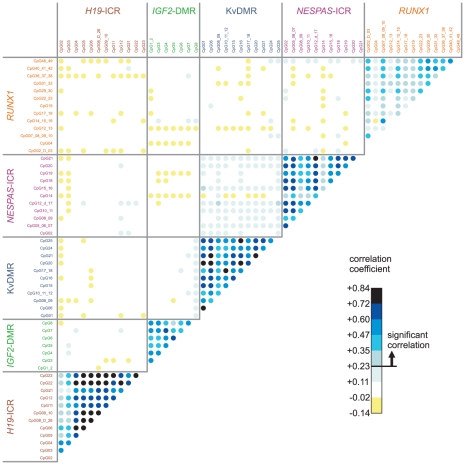
Correlation matrix of DNA methylation levels between all CpG units. The nonparametric Spearman correlation scores between the DNA methylation levels of different CpG units are displayed in a 2D matrix in shades of blue and yellow (for positive and negative values, respectively). All correlation scores above 0.23 are significant (two-tailed, *P*-value<0.0001).

To investigate the relationship between DNA methylation levels at different CpG units measured within an individual, we performed a nonparametric Spearman correlation test and plotted the results in a correlation matrix (Figure 3). Significant correlation scores were obtained for all correlation coefficients above +0.23 (two-tailed, *P*-value<0.0001). As can be clearly seen in Figure 3, the methylation levels *within* each ICR/DMR are highly correlated. Especially in *H19*-ICR, KvDMR and *NESPAS*-ICR strong correlation coefficients were observed (up to 0.84 in KvDMR). In contrast, the *RUNX1* promoter displays more sporadic and random DNA methylation. The correlation *between* different ICRs was much lower. Only between KvDMR and *NESPAS*-ICR a modest, but significant correlation was observed even though these regions are located in different chromosomes (11p15.5 and 20q13.32, respectively). Together, these results demonstrate a considerable degree of variation in the DNA methylation levels at critical regulatory regions in the genome of healthy MZ and DZ twins. In contrast, within an individual, the DNA methylation level at specific CpG sites is highly dependent on the methylation levels of neighbouring sites.

### Intra-twin discordance in DNA methylation profiles

**Figure 4:**
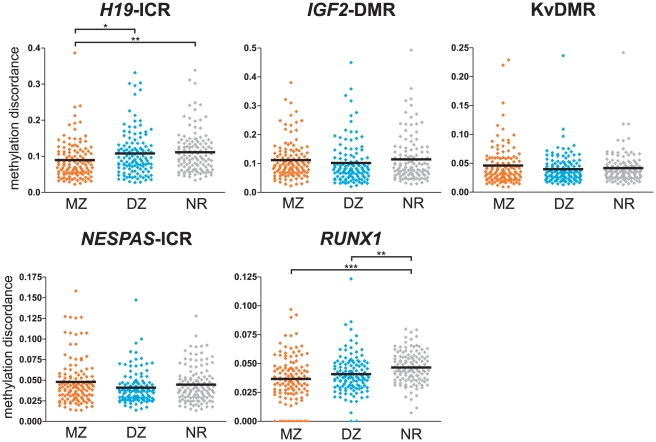
Gene-level analysis of intra-twin discordance in DNA methylation. The discordance in DNA methylation levels was calculated within monozygotic twin pairs, dizygotic twin pairs and between non-related individuals (MZ, DZ and NR, respectively). The methylation differences between individuals were summed over a region and displayed as scatter plots with a horizontal black line representing the group median (see Figures S2, S3, S4, S5, S6 for intra-twin comparisons at each individual CpG unit). Significant differences between groups were identified using the non-parametric Kruskal-Wallis test, followed by a Dunn's multiple comparison test. In *H19*-ICR, the methylation discordance was significantly less in MZ twin pairs than in DZ pairs, or between non-related individuals. Within the *RUNX1* promoter, the non-related individuals displayed the greatest discordance over MZ or DZ twin pairs (*: *P*-value<0.05; **: *P*-value<0.01; ***: *P*-value<0.001).

Next, we investigated the differences in DNA methylation within MZ twin pairs, DZ twin pairs and between non-related individuals to determine if specific loci show discordant methylation between individuals. As DNA methylation levels displayed strong correlations within each region, we averaged the methylation differences per interrogated region (Figure 4; results for individual CpG units can be found as Figures S2, S3, S4, S5, S6). Statistical analysis (Kruskal-Wallis one-way analysis of variance test and a post hoc Dunn's test) revealed significant differences between MZ and DZ methylation discordance for *H19*-ICR and *RUNX1* (*P*-values<0.05). In *H19*-ICR, genetically identical individuals (MZ twins) displayed significantly less methylation discordance than DZ pairs, or non-related individuals suggesting the local genetic sequence may be influencing the methylation status at the *H19*-ICR. For the *RUNX1* promoter the discordance in DNA methylation was also less in MZ or DZ twin pairs compared to non-related individuals. The other interrogated regions did not reveal significant differences in intra-twin discordances, implicating either a lack of genetic or environmental pressure on the DNA methylation levels at these imprinted regions.

### Gain or loss of localised and regional DNA methylation

**Figure 5:**
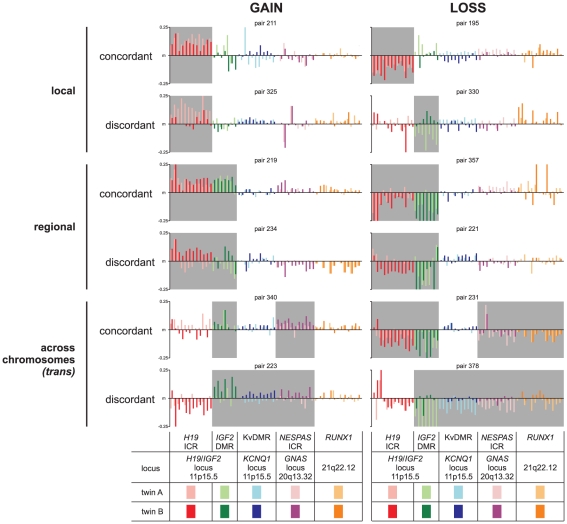
Examples of monozygotic twin pairs with aberrant DNA methylation levels. Typical examples are shown of monozygotic twin pairs harbouring gains or losses in DNA methylation levels relative to the median (m), highlighted in grey. The gains or losses - either concordant or discordant - can be observed within the twin pairs and the aberrations can be local (only one chromosomal region), regional (nearby regions in the genome) or even across different chromosomes (in *trans*). Y-axis shows the DNA methylation difference from the median (m). Colour legends and locus info are shown below the graphs.

Changes in DNA methylation at imprinting control loci or other critical regions in the genome have been clearly associated with many diseases and disease susceptibilities [9], [11], [12], [13], [14], [17], [23], [27], [28], [29]. We therefore investigated the frequency of gain or loss of methylation (GOM or LOM, respectively) in the cohort of adolescent twins, where GOM or LOM calls were determined by 10% deviation from the median DNA methylation levels (Table 1 and Table S2). In addition, we determined whether only one or both individuals of a twin pair displayed the same GOM or LOM, that is were they concordant or discordant in gain or loss of methylation. Interestingly, in most cases LOM or GOM was found to be discordant as only one of the individuals of a twin pair displayed a change, even in MZ twin pairs (Table 1). Still, we observed significantly more MZ than DZ twin pairs with concordant LOM in *H19*-ICR (60 vs. 38; chi-square test, *P*<0.01). Likewise, in *NESPAS*-ICR 22 MZ twins displayed a concordant LOM, whereas no DZ twin pairs showed loss of methylation (chi-square test, *P*<0.001). KvDMR was the only locus that showed discordance for LOM more frequently in MZ twins compared to DZ twins (52 vs. 20; chi-square test, *P*<0.001). Examples of types of variance in DNA methylation levels *within* individual MZ twin pairs, are shown in Figure 5 including *localised concordance*: both individuals of a twin pair displayed losses or gains in DNA methylation in a similar fashion over localised regions, *localised discordance*: only one individual of a twin pair displayed losses or gains in DNA methylation in a discordant fashion over localised regions, *regional concordance* or *discordance*: consistent LOM or GOM across a contiguous region, and *concordance* or *discordance across chromosomes* (*trans*). In conclusion, aberrations in the fine control of DNA methylation levels in imprinted regions are common in MZ and DZ twins and typically concordant and discordant changes occur at local, regional and across chromosomes.

**Table 1:** Loss or gain of methylation (LOM or GOM, respectively: >10% difference from median).

		*H19*-ICR *(%)*	*IGF2*-DMR *(%)*	KvDMR *(%)*	*NESPAS*-ICR *(%)*	*RUNX1 (%)*
**MZ**	concordant for LOM	60 *(2.0)*	74 *(4.3)*	0 *(0.0)*	22 *(0.8)*	56 *(1.8)*
	discordant for LOM	265 *(9.0)*	192 *(11.1)*	52 *(1.9)*	17 *(0.6)*	72 *(2.3)*
	no LOM	2612 *(88.9)*	1464 *(84.6)*	2672 *(98.1)*	2720 *(98.6)*	3047 *(96.0)*
**DZ**	concordant for LOM	38 *(1.3)*	60 *(3.5)*	0 *(0.0)*	0 *(0.0)*	56 *(1.7)*
	discordant for LOM	268 *(9.0)*	195 *(11.4)*	20 *(0.7)*	17 *(0.6)*	72 *(2.2)*
	no LOM	2665 *(89.7)*	1459 *(85.1)*	2735 *(99.3)*	2732 *(99.4)*	3106 *(96.0)*
**chi-square test LOM**		***P*** **<0.01**	**ns**	***P*** **<0.001**	***P*** **<0.001**	**ns**
**MZ**	concordant for GOM	110 *(3.7%)*	24 *(1.4%)*	0 *(0.0%)*	0 *(0.0%)*	4 *(0.1%)*
	discordant for GOM	329 *(11.2%)*	174 *(10.1%)*	30 *(1.1%)*	32 *(1.2%)*	85 *(2.7%)*
	no GOM	2498 *(85.1%)*	1532 *(88.6%)*	2694 *(98.9%)*	2727 *(98.8%)*	3086 *(97.2%)*
**DZ**	concordant for GOM	110 *(3.7%)*	24 *(1.4%)*	0 *(0.0%)*	0 *(0.0%)*	4 *(0.1%)*
	discordant for GOM	329 *(11.1%)*	174 *(10.2%)*	22 *(0.8%)*	32 *(1.2%)*	85 *(2.6%)*
	no GOM	2532 *(85.2%)*	1516 *(88.4%)*	2733 *(99.2%)*	2717 *(98.8%)*	3145 *(97.2%)*
**chi-square test GOM**		**ns**	**ns**	**ns**	**ns**	**ns**

Number (*and percentage*) of observations are displayed in the relevant groups. The chi-square test (3×2; df = 2) was performed to test for significant differences between MZ and DZ twins (see Table S2 for other cut-off values for loss or gain of methylation).

### Influence of genotype on DNA methylation

**Figure 6:**
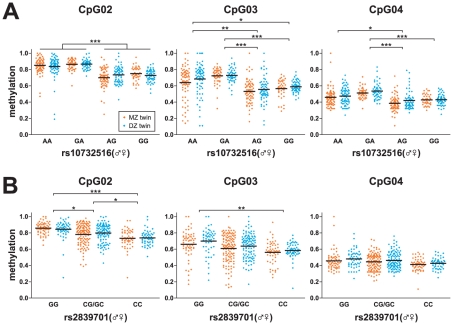
Influence of rs10732516 and rs2839701 on the DNA methylation levels in the *H19*-ICR region. (A) DNA methylation results are sorted on the rs10732516 genotype, where the parent of origin is also taken into account. The rs10732516 [AA] and [GA] genotypes ([♂♀]) display elevated DNA methylation levels compared to [AG] and [GG] genotypes. These differences in DNA methylation are visible throughout the *H19*-ICR locus but are most apparent for CpG sites 2, 3 and 4. (B) Similarly, DNA methylation results are sorted based on the rs2839701 genotype. For CpG sites 2, 3 and 4 in the *H19*-ICR region, the rs2839701 [G] allele is associated with a higher DNA methylation compared to the [C] allele. These results show that both polymorphisms within the *IGF2/H19* region may predict or influence the DNA methylation status in individuals. Significant differences between groups were identified using the non-parametric Kruskal-Wallis test, followed by a Dunn's multiple comparison test (*: *P*-value<0.05; **: *P*-value<0.01; ***: *P*-value<0.001).

**Figure 7:**
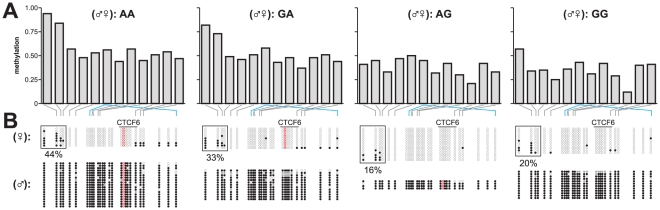
Clonal bisulphite sequencing of *H19*-ICR in individuals with different rs10732516 genotypes. The DNA methylation levels in four samples with different rs10732516 genotypes were further investigated via clonal bisulphite sequencing. (A) MassCleave data for *H19*-ICR of the four genotypes. (B) Clonal bisulphite sequencing analysis of the samples confirmed the genotype of the rs10732516 polymorphism (the [A] allele highlighted in red). In addition, the hypermethylated state of the maternal rs10732516 [A] allele was confirmed. The maternal [A] allele in the [AA] and [GA] individuals displayed higher methylation levels at the 5′-end of the *H19*-ICR than the maternal [G] allele in [AG] or [GG] individuals (44% and 33% versus 16% and 20%, respectively; boxed regions).

As shown in Figure 4, the DNA methylation levels in *H19*-ICR displayed increased similarity when two individuals were more similar at the genetic level (MZ versus DZ twins versus non-related individuals). To further test any influence of genotype on DNA methylation levels, we performed a genotype analysis of a number of single nucleotide polymorphisms (SNPs) in the *H19*/*IGF2* locus (Figure 1A). For one SNP, rs10732516, we were also able to determine allele-specific methylation (Figure S7) [22]. To our surprise, we found that the maternal rs10732516 [A] allele (normally the unmethylated allele in this region), was methylated to greater levels over the entire *H19*-ICR region and most significantly at CpG sites 2, 3 and 4 (Figure 6A; Kruskal-Wallis test with post-hoc a Dunn's test; *P*<0.05). A similar propensity to show hypermethylation was not observed for the maternal rs10732516 [G] allele. Both MZ and DZ individuals displayed a more hypermethylated state in the maternal rs10732516 [A] allele and further investigation of the rs10732516 genotype could fully explain the intra-individual discordance observed in Figure 4 (Kruskal-Wallis test per genotype with post-hoc a Dunn's test was not significant; P>0.05). From the analysis of the other SNPs in the vicinity it became apparent that rs10732516 and rs2839701 are co-inherited as they are part of the same haplotype block; the presence of an rs2839701 [G] allele also marks a hypermethylated state of the *H19*-ICR locus (Figure 6B). As we cannot distinguish the parental origin of the rs2839701 polymorphism, the heterozygote [CG/GC] (♂♀) group displayed a larger variance in DNA methylation compared to [CC] or [GG] homozygotes. Other SNPs however that were tested within the *H19*/*IGF2* locus on 11p15.5 did not correlate with any differential DNA methylation levels for the CpG sites tested (data not shown). We confirmed the increase in methylation of CpG sites at the 5′ end of the *H19*-ICR, using clonal bisulphite sequencing reactions for each genotype (Figure 7). In the rs10732516 [AA] and [GA] (♂♀) genotypes, the maternal alleles displayed elevated methylation levels compared to the [AG] and [GG] genotypes (44% and 33% versus 16% and 20% DNA methylation, respectively).

**Figure 8:**
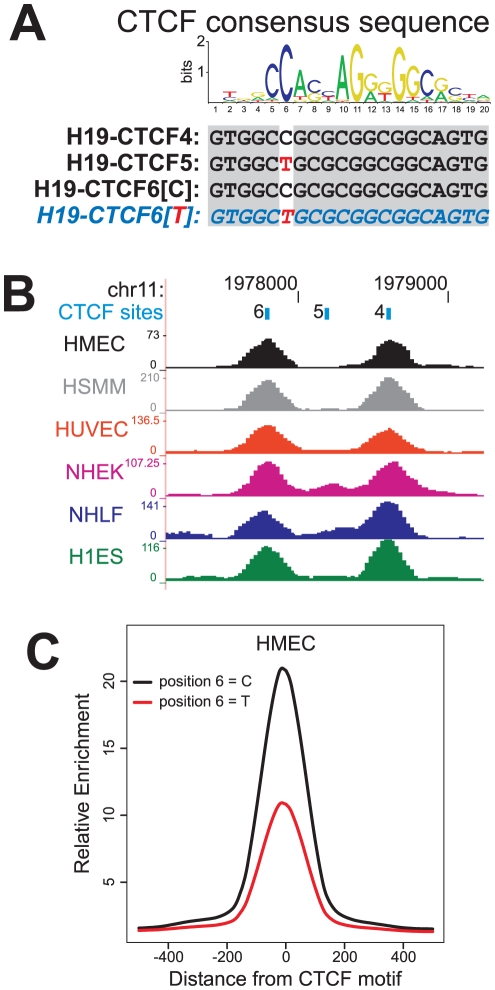
Putative functional implication of rs10732516 on CTCF binding in the *H19*-ICR region. (A) The Position Weight Matrix (PWM) of the CTCF consensus sequence as determined by Cuddapah *et al.* (2009) [44] harbours a strong preference for a cytosine residue at position 6 of the PWM. Within the region upstream the *H19* gene, this cytosine is present in CTCF binding site numbers 4 and 6 (for the rs10732516 [G] allele) [45]. The CTCF binding site number 5 within the *H19* region harbours a thymidine at position 6, and is identical in sequence to CTCF binding site number 6 for the rs10732516 [A] allele (CTCF sequences are on the minus strand). (B) Analysis of CTCF binding at the *H19* promoter region covering CTCF binding sites 4, 5 and 6 in six normal cell lines identified site number 5 to have minimal binding affinity within the region (ChIP-seq data from the ENCODE Chromatin Group at the Broad Institute [25]; UCSC Genome Browser; HMEC: human mammary epithelial cells; HSMM: normal human skeletal muscle myoblasts; HUVEC: human umbilical vein endothelial cells; NHEK: normal human epidermal keratinocytes; NHLF: normal human lung fibroblasts; H1ES: human embryonic stem cell line H1). (C) Effect of cytosine versus thymidine at position 6 of the PWM on the average enrichment score near CTCF sites within the genome of HMEC cells. Positions matching the PWM with 80% or greater sequence identity were identified and the average enrichment scores near these sites were determined −500 to +500 base pairs relative to the CTCF motif. Results for the other cell lines can be found as Figure S8. These combined results suggest a strong influence of the rs10732516 genotype on the binding affinity of CTCF at this position in the *H19*-ICR.

Notably, the rs10732516 polymorphism directly overlaps with the 6^th^ CTCF binding site within this region (Figure 7, 8A), providing a possible mechanistic link between the nature of the SNP and the preponderance for methylation. When comparing the CTCF binding strengths at the various CTCF binding sites in the region in normal cell lines (ENCODE ChIP-seq data from the Chromatin Group at Broad Institute) [25] we noted the results for the fifth CTCF binding site are markedly reduced compared to the other binding sites in the region (Figure 8B). The sequence of the fifth CTCF site is 100% identical to the binding site containing the rs10732516 [A] polymorphism (Figure 8A). Furthermore, genome-wide analyses of the binding strength at locations where either a cytosine or thymidine is present at position 6 of the consensus CTCF sequence also revealed a drastic reduction of CTCF enrichment scores in case of a thymidine at this position (Figure 8C and Figure S8). This data suggests that in the *H19*-ICR region, the genotype of the underlying sequence (in particular the rs10732516 polymorphism) may have a substantial impact on the determination of the DNA methylation status of the locus.

## Discussion

Over the past decades, the importance of studies on monozygotic twins has become increasingly apparent. These studies have turned out to be very effective in addressing key questions in human biology, ranging from the genetics of social behaviour, and nature versus nurture dilemmas, to the heritability of phenotypic variation and disorders [9], [30], [31], [32]. More recently, DNA methylation levels have also become an important research topic in twins [33], [34], [35], [36], [37], [38]. In this report, we studied mono- (MZ) and dizygotic (DZ) twins to address the fundamental question of the impact of the genome on the degree of variation of the epigenome by surveying the DNA methylation levels in the imprinting control regions *H19*-ICR, *IGF2*-DMR, KvDMR and *NESPAS*-ICR and in the sporadically methylated *RUNX1*/*AML1* promoter, as examples of genes exquisitely controlled by DNA methylation. Using genomic DNA from whole blood we interrogated the DNA methylation levels at 83 CpG sites present in five regions in 256 healthy adolescent twins pairs, using a highly sensitive and quantitative mass spectrometric approach [22].

We show that even in MZ twins, a considerable degree of variability exists in DNA methylation patterns, and we propose that this may impact on variability in gene expression and possible differences in disease susceptibility. Indeed, it has been shown that deregulation of the imprinting status is linked to a number of disorders [9], [11], [12], [13], [14], [17], [23], [27], [28], [29], [39]. Conversely, the observed variability could imply that the DNA methylation levels at these positions in the genome are less critical in blood cells or in adolescent life.

The intra-twin discordance analysis revealed MZ twins display more similar DNA methylation levels in *H19*-ICR and the *RUNX1* promoter compared to DZ twins or non-related individuals. Overall, strong correlations in DNA methylation were observed between CpG sites of the same region. Similar CpG site-to-site dependencies in DNA methylation patterns have been suggested previously [40], [41]. These data support the key role of the (local) genetic sequence or genomic context in the regulation of DNA methylation levels [33], [34], [36], [38]. Interestingly, the observed correlations did not extend into other regions, not even between the closely related *IGF2*-DMR and *H19*-ICR, which are part of the same imprinting locus with DNA methylation marks present on the same parental allele. This is in contrast to patients with Beckwith-Wiedemann or Silver-Russell syndrome where both the *H19*-ICR and *IGF2*-DMR display a gain or loss of methylation, respectively [42]. The independent behaviour of the regions tested suggests no major global, chromosomal or parental regulation of DNA methylation levels.

Interestingly, the analysis of gains or losses in DNA methylation revealed frequent concordant losses in *H19*-ICR and *NESPAS*-ICR in both individuals of an MZ twin pair, more frequent than in DZ twin pairs, although some discordance in MZ twins was also observed for KvDMR. The concordant deregulation further implicates a genetic component to be involved in controlling the DNA methylation levels in these regions. Still, most cases of deregulation (loss or gain) of DNA methylation are found to be discordant, affecting only one individual of a twin pair. Typically, these aberrations in the fine control of DNA methylation levels can occur at the local or regional level, or even across chromosomes, in *trans*. In about 15% of individuals tested the *IGF2*-DMR displayed a loss of methylation (LOM), compared to 11% with a gain in DNA methylation (GOM). A previous study reported about 10% of healthy individuals to display LOM and proposed that this may be a predictive marker of risk for developing colorectal cancer (CRC) [9]. To date, no cases of CRC are known within our adolescent twin cohort.

Finally, the analysis of single nucleotide polymorphisms near the interrogated imprinting control regions yielded compelling evidence for a genetic impact on DNA methylation levels at imprinted regions. In the *IGF2*/*H19* domain, a striking correlation was found between the maternal rs10732516 [A/G] polymorphism - as well as the co-inherited rs2839701 [G/C] polymorphism - and the DNA methylation state of the *H19*-ICR locus, where both the maternal rs10732516 [A] allele and rs2839701 [G] allele are linked to a hypermethylated state. The maternal *H19*-ICR allele is generally unmethylated allowing CTCF to bind to its seven binding sites in the region. However, the presence of the rs10732516 [A] allele changes the sixth CTCF binding site in the region, which, according to Takai *et al* [23], is the key CTCF binding site regulating the expression of IGF2. In humans, it has been demonstrated that loss of differential methylation at this site correlates with loss of imprinting (LOI) in Wilms' tumours [27], bladder cancer [23], colon cancer [28], [29] and osteosarcoma [39]. Our detailed DNA methylation data of this region combined with the reinterpretation of results of genome-wide CTCF binding studies [25] suggests that the rs10732516 [A/G] polymorphism may directly affect the binding affinity of CTCF to its target sequence. This may also implicate a functional link between the maternal rs10732516 and the methylation status of the (maternal) allele. Future experiments should yield more insight into the functional link between the SNP or local genotype and the deregulated DNA methylation levels. Interestingly, it is not the CpG sites immediately adjacent to rs10732516 that display the most aberrant DNA methylation state. The CpG site with the strongest deregulation is located 195 base pairs (or 15 CpG sites) upstream of the SNP, proximal to the *H19* gene. To exclude the possibility of bias in our data introduced by genomic polymorphisms or mutations within the primer binding sequences, DNA sequencing analysis of all interrogated regions were performed. No mutations or genomic events were observed that could account for a bias in the DNA methylation assays. To our knowledge, this is the first time a single nucleotide polymorphism is associated with parental specific methylation differences in the IGF2/H19 locus. For the *RUNX1* promoter, the local genotype near the *RUNX1* gene may also be involved in the methylation discordance observed in DZ twin pairs and between non-related individuals. Further research will be necessary to resolve this question.

In conclusion, we have shown that a considerable degree of variation in the DNA methylation at critically important regions in the genome exists, even in genetically identical individuals, and we have identified a clear example of the impact of the underlying genetic sequence on potential perturbations of the epigenome.

## Supporting Information

Figure S1 **Details of interrogated CpG sites and regions.** Chromosomal coordinates and fragmentation patterns are given for each assay. Above each graph the numbering of the CpG sites is shown and below each graph the names of the interrogated fragments are given between brackets. Interrogated and not-interrogated CpG sites are shown as red and grey dots, respectively. The CpG site containing rs10732516 [A/G] is shown in blue, while the rs45596642 [A/T] is highlighted in green. The vertical black lines in the graphs represent RNase A cleavage sites. Figures have been generated using the ampliconReport R-script [22].
(EPS)

Figure S2 **Intra-twin discordance in DNA methylation in** ***H19*** **-ICR per CpG unit.** The discordance in DNA methylation levels was calculated within MZ twin pairs, DZ twin pairs and between non-related individuals (MZ, DZ and NR, respectively). Significant differences between groups were identified using the non-parametric Kruskal-Wallis test, followed by a Dunn's multiple comparison test (*: *P*-value<0.05; **: *P*-value<0.01).
(EPS)

Figure S3 **Intra-twin discordance in DNA methylation in** ***IGF2*** **-DMR per CpG unit.** The discordance in DNA methylation levels was calculated within MZ twin pairs, DZ twin pairs and between non-related individuals (MZ, DZ and NR, respectively). Significant differences between groups were identified using the non-parametric Kruskal-Wallis test, followed by a Dunn's multiple comparison test (*: *P*-value<0.05; **: *P*-value<0.01).
(EPS)

Figure S4 **Intra-twin discordance in DNA methylation in KvDMR per CpG unit.** The discordance in DNA methylation levels was calculated within MZ twin pairs, DZ twin pairs and between non-related individuals (MZ, DZ and NR, respectively). Significant differences between groups were identified using the non-parametric Kruskal-Wallis test, followed by a Dunn's multiple comparison test (*: *P*-value<0.05; **: *P*-value<0.01).
(EPS)

Figure S5 **Intra-twin discordance in DNA methylation in** ***NESPAS*** **-DMR per CpG unit.** The discordance in DNA methylation levels was calculated within MZ twin pairs, DZ twin pairs and between non-related individuals (MZ, DZ and NR, respectively). Significant differences between groups were identified using the non-parametric Kruskal-Wallis test, followed by a Dunn's multiple comparison test (*: *P*-value<0.05; **: *P*-value<0.01).
(EPS)

Figure S6 **Intra-twin discordance in DNA methylation in the** ***RUNX1*** **promoter per CpG unit.** The discordance in DNA methylation levels was calculated within MZ twin pairs, DZ twin pairs and between non-related individuals (MZ, DZ and NR, respectively). Significant differences between groups were identified using the non-parametric Kruskal-Wallis test, followed by a Dunn's multiple comparison test (*: *P*-value<0.05; **: *P*-value<0.01).
(EPS)

Figure S7 **Simultaneous SNP genotyping and analysis of the allele-specific methylation status from MassCleave spectra.** Detailed example of four different genotypes analyzed for the DNA methylation status of *H19-ICR*. The SNP status for rs10732516 could easily be determined, as this polymorphism changes the positions of spectral peaks. The peaks that change also contain a number of CpG sites, so the methylation status associated with the polymorphism could be deduced simultaneously. The unmethylated rs10732516 [G] allele is visible as a peak at 5352.40 Da, while the methylated rs10732516 [G] allele forms a peak at 5416.40 Da. *) The peaks for the rs10732516 [A] allele (2878.82 and 2894.82 Da) overlap with peaks of another fragment of *H19-ICR* (CpG7) resulting in more subtle spectral changes at these positions. As the [A] allele has introduced a novel cleavage site for the RNase A, only one CpG site is associated with this SNP. The grey crossed-out circles indicate CpG sites not interrogated in the assay. Figure was adapted from Coolen, *et al.* (2007) [22].
(EPS)

Figure S8 **Effect of cytosine versus thymidine at position 6 of the Position Weight Matrix (PWM) for CTCF binding sites on the average enrichment score near CTCF motifs within the genome of six normal cell lines.** Positions matching the PWM with 80% or greater sequence identity were identified and the average enrichment scores near these sites were determined −500 to +500 base pairs relative to the CTCF motif. The mean enrichment scores for the PWM with a thymidine at position 6 are about half of the scores with a cytosine at this position, in all cell lines tested (HMEC: human mammary epithelial cells; HSMM: normal human skeletal muscle myoblasts; HUVEC: human umbilical vein endothelial cells; NHEK: normal human epidermal keratinocytes; NHLF: normal human lung fibroblasts; H1ES: human embryonic stem cell line H1).
(EPS)

Table S1 **Primers and PCR conditions.**
(PDF)

Table S2 **Loss and gain of methylation in twins (various cut-offs for difference from median).**
(PDF)
