# Supplementary material for: Impact of the Genome on the Epigenome Is Manifested in DNA Methylation Patterns of Imprinted Regions in Monozygotic and Dizygotic Twins
Source: PLoS One. 2011 Oct 3;6(10):e25590. doi: 10.1371/journal.pone.0025590 (PMC3184992; doi:10.1371/journal.pone.0025590)
Supplement: Table S1 — Primers and PCR conditions. (PDF) [file pone.0025590.s009.pdf]

Table S1. Primers and PCR conditions

Sequenom primers							
**Region**	**Primer**	**Sequence (5'-3')**	**Coordinates *)**	**Strand**	**Span**	**Ta(1)**	**Ta(2)**
H19-ICR	tag-H19-FW	AGGAAGAGAG**GGAAAATGTAAGATTTTGGTGGAATAT**	chr11:1,977,501-1,977,965	(+)	465 bp	59.1	68
	T7-H19-RV	CAGTAATACGACTCACTATAGGGAGAAGGCT**TCCCAATTCCATAAATAATAAAAATCTC**					
IGF2-DMR	tag-IGF2-FW	AGGAAGAGAG**GATTTATTTAGGGTGGTGTTTGTGG**	chr11:2,125,822-2,126,153	(-)	332 bp	62.8	70
	T7-IGF2-RV	CAGTAATACGACTCACTATAGGGAGAAGGCT**AAAAAATTTACCTAAAAAAAACTTCCC**					
KvDMR	tag-KvDMR-FW	AGGAAGAGAG**TTTGGTAGGATTTTGTTGAGGAGTTT**	chr11:2,677,737-2,678,041	(+)	305 bp	62.8	70
	T7-KvDMR-RV	CAGTAATACGACTCACTATAGGGAGAAGGCT**CTCACACCCAACCAATACCTCATAC**					
NESPAS-ICR	tag-NESPAS-FW	AGGAAGAGAG**GGGTTAGTTATTGGGTAGGGGTTATGT**	chr20:56,859,080-56,859,449	(-)	370 bp	62.8	70
	T7-NESPAS-RV	CAGTAATACGACTCACTATAGGGAGAAGGCT**CCTCCCAACCCTAAAAATCTTCTTACT**					
RUNX1	tag-RUNX1-FW	AGGAAGAGAG**YGGGGTGGATGAGAGGTTT**	chr21:35,183,623-35,184,067	(-)	445 bp	52.9	68
	T7-RUNX1-RV	CAGTAATACGACTCACTATAGGGAGAAGGCT**ACCAACRAAAAATTTCCTAATCAAA**					

***) chromosomal positions according to the UCSC Genome Browser - March 2006 assembly**

PCR on bisulphite-treated gDNA:
1x: 95°C for 2 min; 5x: 95°C for 20 s, Ta(1) for 30s, 72°C for 1 min; 40x: 95°C for 20 s, Ta(2) for 30s, 72°C for 1 min; 1x 72°C for 5 min.

Genomic primers							
**Region**	**Primer**	**Sequence (5'-3')**	**Coordinates *)**	**Strand**	**Span**	**Ta(1)**	**Ta(2)**
rs10732516	H19-rs10732516-FW	**ACGCGTGGCTTGGGTGA**	chr11:1977692-1977869	NA	178 bp	60	NA
	H19-rs10732516-RV	**CATGGGTATTTCTGGAGGCTTCT**					
rs45596642	NESPAS-rs45596642-FW	**CCTCTTCGGGCGTTCCAA**	chr20:56859322-56859518	NA	197 bp	60	NA
	NESPAS-rs45596642-RV	**CACTTGAGCATCCACTGAATGG**					

***) chromosomal positions according to the UCSC Genome Browser - March 2006 assembly**

PCR on gDNA:
1x: 95°C for 2 min; 40x: 95°C for 20 s, Ta(1) for 30s, 72°C for 1 min; 1x 72°C for 5 min.
