# Supplementary material for: Impact of the Genome on the Epigenome Is Manifested in DNA Methylation Patterns of Imprinted Regions in Monozygotic and Dizygotic Twins
Source: PLoS One. 2011 Oct 3;6(10):e25590. doi: 10.1371/journal.pone.0025590 (PMC3184992; doi:10.1371/journal.pone.0025590)
Supplement: Table S2 — Loss and gain of methylation in twins (various cut-offs for difference from median). (PDF) [file pone.0025590.s010.pdf]

Table S2. Loss or gain of methylation (LOM or GOM, respectively)

> 2.5% difference from median		*H19-ICR*	*IGF2-DMR*	KvDMR	*NESPAS-ICR*	*RUNX1*
MZ	LOM in twin pairs	454 *15.5%*	292 *16.9%*	228 *8.4%*	196 *7.1%*	204 *6.4%*
	LOM not in twin pairs	669 *22.8%*	355 *20.5%*	416 *15.3%*	456 *16.5%*	334 *10.5%*
	no LOM	1814 *61.8%*	1083 *62.6%*	2080 *76.4%*	2107 *76.4%*	2637 *83.1%*
	**chi-square test**	**ns**	**ns**	***P* < 0.001**	**ns**	**ns**
DZ	LOM in twin pairs	450 *15.1%*	278 *16.2%*	148 *5.4%*	174 *6.3%*	198 *6.1%*
	LOM not in twin pairs	671 *22.6%*	362 *21.1%*	445 *16.2%*	457 *16.6%*	337 *10.4%*
	no LOM	1850 *62.3%*	1074 *62.7%*	2162 *78.5%*	2118 *77.0%*	2699 *83.5%*
MZ	GOM in twin pairs	458 *15.6%*	248 *14.3%*	188 *6.9%*	198 *7.2%*	266 *8.4%*
	GOM not in twin pairs	696 *23.7%*	390 *22.5%*	499 *18.3%*	475 *17.2%*	557 *17.5%*
	no GOM	1783 *60.7%*	1092 *63.1%*	2037 *74.8%*	2086 *75.6%*	2352 *74.1%*
	**chi-square test**	**ns**	**ns**	***P* < 0.001**	**ns**	**ns**
DZ	GOM in twin pairs	458 *15.4%*	248 *14.5%*	152 *5.5%*	200 *7.3%*	266 *8.2%*
	GOM not in twin pairs	696 *23.4%*	390 *22.8%*	450 *16.3%*	483 *17.6%*	561 *17.3%*
	no GOM	1817 *61.2%*	1076 *62.8%*	2153 *78.1%*	2066 *75.2%*	2407 *74.4%*

> 5.0% difference from median		*H19-ICR*	*IGF2-DMR*	KvDMR	*NESPAS-ICR*	*RUNX1*
MZ	LOM in twin pairs	192 *6.5%*	180 *10.4%*	22 *0.8%*	50 *1.8%*	88 *2.8%*
	LOM not in twin pairs	549 *18.7%*	290 *16.8%*	245 *9.0%*	172 *6.2%*	155 *4.9%*
	no LOM	2196 *74.8%*	1260 *72.8%*	2457 *90.2%*	2537 *92.0%*	2932 *92.3%*
	**chi-square test**	**ns**	**ns**	***P* < 0.001**	***P* < 0.001**	**ns**
DZ	LOM in twin pairs	180 *6.1%*	166 *9.7%*	12 *0.4%*	28 *1.0%*	86 *2.7%*
	LOM not in twin pairs	553 *18.6%*	297 *17.3%*	142 *5.2%*	172 *6.3%*	155 *4.8%*
	no LOM	2238 *75.3%*	1251 *73.0%*	2601 *94.4%*	2549 *92.7%*	2993 *92.5%*
MZ	GOM in twin pairs	250 *8.5%*	108 *6.2%*	24 *0.9%*	16 *0.6%*	52 *1.6%*
	GOM not in twin pairs	590 *20.1%*	308 *17.8%*	190 *7.0%*	193 *7.0%*	313 *9.9%*
	no GOM	2097 *71.4%*	1314 *76.0%*	2510 *92.1%*	2550 *92.4%*	2810 *88.5%*
	**chi-square test**	**ns**	**ns**	***P* < 0.01**	**ns**	**ns**
DZ	GOM in twin pairs	250 *8.4%*	108 *6.3%*	26 *0.9%*	16 *0.6%*	52 *1.6%*
	GOM not in twin pairs	590 *19.9%*	308 *18.0%*	155 *5.6%*	195 *7.1%*	314 *9.7%*
	no GOM	2131 *71.7%*	1298 *75.7%*	2574 *93.4%*	2538 *92.3%*	2868 *88.7%*

> 7.5% difference from median		*H19-ICR*	*IGF2-DMR*	KvDMR	*NESPAS-ICR*	*RUNX1*
MZ	LOM in twin pairs	100 *3.4%*	120 *6.9%*	0 *0.0%*	28 *1.0%*	58 *1.8%*
	LOM not in twin pairs	418 *14.2%*	242 *14.0%*	136 *5.0%*	50 *1.8%*	85 *2.7%*
	no LOM	2419 *82.4%*	1368 *79.1%*	2588 *95.0%*	2681 *97.2%*	3032 *95.5%*
	**chi-square test**	**ns**	**ns**	***P* < 0.001**	***P* < 0.001**	**ns**
DZ	LOM in twin pairs	82 *2.8%*	106 *6.2%*	2 *0.1%*	6 *0.2%*	58 *1.8%*
	LOM not in twin pairs	422 *14.2%*	247 *14.4%*	51 *1.9%*	50 *1.8%*	85 *2.6%*
	no LOM	2467 *83.0%*	1361 *79.4%*	2702 *98.1%*	2693 *98.0%*	3091 *95.6%*
MZ	GOM in twin pairs	182 *6.2%*	52 *3.0%*	2 *0.1%*	4 *0.1%*	18 *0.6%*
	GOM not in twin pairs	453 *15.4%*	247 *14.3%*	88 *3.2%*	84 *3.0%*	170 *5.4%*
	no GOM	2302 *78.4%*	1431 *82.7%*	2634 *96.7%*	2671 *96.8%*	2987 *94.1%*
	**chi-square test**	**ns**	**ns**	***P* < 0.001**	**ns**	**ns**
DZ	GOM in twin pairs	182 *6.1%*	52 *3.0%*	4 *0.1%*	4 *0.1%*	18 *0.6%*
	GOM not in twin pairs	453 *15.2%*	247 *14.4%*	60 *2.2%*	84 *3.1%*	171 *5.3%*
	no GOM	2336 *78.6%*	1415 *82.6%*	2691 *97.7%*	2661 *96.8%*	3045 *94.2%*

Table S2. Loss or gain of methylation (LOM or GOM, respectively), continued

> 10.0% difference from median		*H19-ICR*	*IGF2-DMR*	KvDMR	*NESPAS-ICR*	*RUNX1*
MZ	LOM in twin pairs	60 *2.0%*	74 *4.3%*	0 *0.0%*	22 *0.8%*	56 *1.8%*
	LOM not in twin pairs	265 *9.0%*	192 *11.1%*	52 *1.9%*	17 *0.6%*	72 *2.3%*
	no LOM	2612 *88.9%*	1464 *84.6%*	2672 *98.1%*	2720 *98.6%*	3047 *96.0%*
	**chi-square test**	***P*** **< 0.01**	**ns**	***P*** **< 0.001**	***P*** **< 0.001**	**ns**
DZ	LOM in twin pairs	38 *1.3%*	60 *3.5%*	0 *0.0%*	0 *0.0%*	56 *1.7%*
	LOM not in twin pairs	268 *9.0%*	195 *11.4%*	20 *0.7%*	17 *0.6%*	72 *2.2%*
	no LOM	2665 *89.7%*	1459 *85.1%*	2735 *99.3%*	2732 *99.4%*	3106 *96.0%*
MZ	GOM in twin pairs	110 *3.7%*	24 *1.4%*	0 *0.0%*	0 *0.0%*	4 *0.1%*
	GOM not in twin pairs	329 *11.2%*	174 *10.1%*	30 *1.1%*	32 *1.2%*	85 *2.7%*
	no GOM	2498 *85.1%*	1532 *88.6%*	2694 *98.9%*	2727 *98.8%*	3086 *97.2%*
	**chi-square test**	**ns**	**ns**	**ns**	**ns**	**ns**
DZ	GOM in twin pairs	110 *3.7%*	24 *1.4%*	0 *0.0%*	0 *0.0%*	4 *0.1%*
	GOM not in twin pairs	329 *11.1%*	174 *10.2%*	22 *0.8%*	32 *1.2%*	85 *2.6%*
	no GOM	2532 *85.2%*	1516 *88.4%*	2733 *99.2%*	2717 *98.8%*	3145 *97.2%*

> 12.5% difference from median		*H19-ICR*	*IGF2-DMR*	KvDMR	*NESPAS-ICR*	*RUNX1*
MZ	LOM in twin pairs	42 *1.4%*	46 *2.7%*	0 *0.0%*	22 *0.8%*	0 *0.0%*
	LOM not in twin pairs	190 *6.5%*	147 *8.5%*	24 *0.9%*	7 *0.3%*	0 *0.0%*
	no LOM	2705 *92.1%*	1537 *88.8%*	2700 *99.1%*	2730 *98.9%*	3175 *100.0%*
	**chi-square test**	***P*** **< 0.001**	**ns**	***P*** **< 0.05**	***P*** **< 0.001**	**ns**
DZ	LOM in twin pairs	18 *0.6%*	32 *1.9%*	0 *0.0%*	0 *0.0%*	0 *0.0%*
	LOM not in twin pairs	192 *6.5%*	149 *8.7%*	15 *0.5%*	7 *0.3%*	0 *0.0%*
	no LOM	2761 *92.9%*	1533 *89.4%*	2740 *99.5%*	2742 *99.7%*	3234 *100.0%*
MZ	GOM in twin pairs	74 *2.5%*	10 *0.6%*	0 *0.0%*	0 *0.0%*	2 *0.1%*
	GOM not in twin pairs	254 *8.6%*	117 *6.8%*	20 *0.7%*	24 *0.9%*	59 *1.9%*
	no GOM	2609 *88.8%*	1603 *92.7%*	2704 *99.3%*	2735 *99.1%*	3114 *98.1%*
	**chi-square test**	**ns**	**ns**	**ns**	**ns**	**ns**
DZ	GOM in twin pairs	74 *2.5%*	10 *0.6%*	0 *0.0%*	0 *0.0%*	2 *0.1%*
	GOM not in twin pairs	254 *8.5%*	117 *6.8%*	16 *0.6%*	24 *0.9%*	59 *1.8%*
	no GOM	2643 *89.0%*	1587 *92.6%*	2739 *99.4%*	2725 *99.1%*	3173 *98.1%*

> 15.0% difference from median		*H19-ICR*	*IGF2-DMR*	KvDMR	*NESPAS-ICR*	*RUNX1*
MZ	LOM in twin pairs	30 *1.0%*	34 *2.0%*	0 *0.0%*	22 *0.8%*	0 *0.0%*
	LOM not in twin pairs	124 *4.2%*	104 *6.0%*	11 *0.4%*	4 *0.1%*	0 *0.0%*
	no LOM	2783 *94.8%*	1592 *92.0%*	2713 *99.6%*	2733 *99.1%*	3175 *100.0%*
	**chi-square test**	***P*** **< 0.001**	***P*** **< 0.01**	**ns**	***P*** **< 0.001**	**ns**
DZ	LOM in twin pairs	6 *0.2%*	20 *1.2%*	0 *0.0%*	0 *0.0%*	0 *0.0%*
	LOM not in twin pairs	124 *4.2%*	105 *6.1%*	13 *0.5%*	4 *0.1%*	0 *0.0%*
	no LOM	2841 *95.6%*	1589 *92.7%*	2742 *99.5%*	2745 *99.9%*	3234 *100.0%*
MZ	GOM in twin pairs	62 *2.1%*	6 *0.3%*	0 *0.0%*	0 *0.0%*	0 *0.0%*
	GOM not in twin pairs	180 *6.1%*	79 *4.6%*	17 *0.6%*	8 *0.3%*	32 *1.0%*
	no GOM	2695 *91.8%*	1645 *95.1%*	2707 *99.4%*	2751 *99.7%*	3143 *99.0%*
	**chi-square test**	**ns**	**ns**	**ns**	**ns**	**ns**
DZ	GOM in twin pairs	62 *2.1%*	6 *0.4%*	0 *0.0%*	0 *0.0%*	0 *0.0%*
	GOM not in twin pairs	180 *6.1%*	79 *4.6%*	11 *0.4%*	8 *0.3%*	32 *1.0%*
	no GOM	2729 *91.9%*	1629 *95.0%*	2744 *99.6%*	2741 *99.7%*	3202 *99.0%*
